# Mechanism of cargo sorting into small extracellular vesicles

**DOI:** 10.1080/21655979.2021.1977767

**Published:** 2021-10-18

**Authors:** Yiwen Chen, Yuxue Zhao, Yiqian Yin, Xiaonan Jia, Lingxiang Mao

**Affiliations:** a Department of Laboratory Medicine, The Affiliated People’s Hospital, Jiangsu University, Zhenjiang, China; b Department of Immunology, Jiangsu Key Laboratory of Laboratory Medicine, School of Medicine, Jiangsu University, Zhenjiang, China

**Keywords:** Small extracellular vesicle, exosomes, sorting, protein, microRNA

## Abstract

Extracellular vesicles (EVs) are special membranous structures released by almost every cell type that carry and protect some biomolecules from being degraded. They transport important signaling molecules involved in cell communication, migration, and numerous physiological processes. EVs can be categorized into two main types according to their size: i) small extracellular vesicles (sEVs), such as exosomes (30–150 nm), released from the fusion of multivesicular bodies (MVBs) with the plasma membrane, and ii) large EVs, such as microvesicles (100–1000 nm). These are no longer considered a waste product of cells, but regulators of intercellular communication, as they can transport specific repertoires of cargos, such as proteins, lipids, and nucleic acids to receptor cells to achieve cell-to-cell communication. This indicates the existence of different mechanisms, which controls the cargos sorting into EVs. This review mainly gives a description about the biological roles of the cargo and the sorting mechanisms of sEVs, especially exosomes.

## 1. Introduction

Extracellular vesicles (EVs) are particles naturally released from various types of cells that are delimited by a lipid bilayer and cannot replicate. Exosomes are small EVs (sEVs, 30–200 nm) derived from the luminal membranes of multivesicular bodies (MVBs) and constitutively released by fusion with the cell membrane [1,2]. It was reported that exosomes originate from the inward budding of the endosomal membrane, thus forming intraluminal vesicles (ILVs), which then enter multivesicular bodies. Subsequently, when MVBs finally fuse with the plasma membrane, the exosomes are secreted into the extracellular space [3]. Exosomes are surrounded by a lipid bilayer structure naturally found in biological fluids, including blood, urine, and saliva et al. Because of the protection of the double-membrane structure, exosomes can contain a wealth of cargos, including diverse proteins, lipids, and nucleic acids [4–6]. Exosomes play a crucial role in cell-to-cell communication via the transportation of those bioactive cargos from the donor to the receptor cells. Exosomes have been demonstrated as carriers for transport of exogenous or endogenous cargos for several therapeutic purposes, followed by the difference of the cargo between the exosomes and the corresponding cells of origin indicating the sorting mechanisms, which it is of significance to understand [7].

The aim of this review is to summarize some classical mechanisms of soring proteins, nucleic acids or lipids in to sEVs, especially exosomes, so as to better understand the significance of these cargos which been sorted in to sEVs, as well as the way of transferring some functional cargos to exosomes to contribute to the disease prediction or treatment.

## 2 Protein cargo in exosomes

### 2.1 Protein cargo types in exosomes

There are probably two types of proteins in the exosomes, the one that can be secreted by almost all exosomes regardless of their types, so these proteins may serve as the marker of exosomes. The most common proteins of exosomes include tetraspanins, cytoplasmic proteins, membrane-binding proteins, and annexins. Among them, different types of proteins play different roles. For instance, tetraspanins are transmembrane proteins that possess four transmembrane domains, exosomes are enriched in the tetraspanins such as CD9, CD63, CD81, and they play a crucial role in sorting, membrane trafficking, and can be positive protein markers of exosomes [8]. Rab families are common cytoplasmic proteins, which are the largest families belonging with small GTPases, and can interact with some proteins to regulate vesicle fusion. Exosomes also enriched with annexins, including annexin 1,2,4,5,6,7,11, etc., which could participate in membrane exchange and fusion [9,10].

Other types of proteins in exosomes specifically depend on their parental cells. Exosomes originating from different host cells may possess various different proteins playing unique roles. Exosomes derived from cancer cells may provide a sign for the detection of early cancer due to the specific cargo they carry [11,12]. For example, exosomal glycoprotein A33, a type I membrane protein, originates only from colon epithelial cells, and may serve as an immunomarker for colorectal carcinoma (CRC) [13]; exosomal immune molecules, such as MHC molecules, originate from antigen presenting cells and are associated with specific immune responses [14]. Meanwhile, several researchers have shown that some important markers are released in culture that may also be present in the exosome content. For example, the amount of CD45 and CD30 molecules increased in cell culture medium after TNF-αtreatment without the destruction of the cell membrane, which indicates exosomal release mechanism by which TNF-a induces release of CD30 and CD45 [15]. Furthermore, lactate dehydrogenase (LDH), a tumor biomarker, could be released from different types of cancer cells in vitro possibly through the exosomes, which may be used for early clinical tumor diagnosis [16]. Analyzing specific protein cargos in the exosome can serve to identify novel biomarkers for some diseases, especially cancer, as overexpressed markers can be detected and analyzed in exosomes [17].

### 2.2 Sorting mechanism of protein cargos

Ubiquitination is a post-translational modification (PTMs), initially known to be a measure of membrane protein degradation. These unwanted or misfolding proteins can be ubiquitinated to become the target of proteasomal degradation, or they can be delivered to Golgi, where the misfolding protein can also be recognized and sent back to the endoplasmic reticulum through coat protein I (COPI) vesicles, or be ubiquitinated at the Golgi, and finally be degraded though the lysosomal pathway [18]. However, this also plays essential part in protein cargo sorting via cooperating with the endosomal sorting complex required for the transport (ESCRT) complex. The ESCRT pathway is not only a key regulator of MVB biogenesis, but also can sort ubiquitinylated protein into exosomes via an exact division of labor of ESCRT complexes, and some molecules of the ESCRT complex, such as ALG-2-interacting protein X (Alix), Vacuolar protein sorting 4(Vps4) also participate in the protein sorting at different stages. In addition to ubiquitination, other PTMs like SUMOylation, Neddylation, etc. are also included in the sorting of proteins into sEVs, probably because they all possess the ubiquitin-like structure. Furthermore, other mechanisms exist, the lipid raft mainly sorts the lipoproteins to the exosomes, regardless of the ESCRT pathway; some tetraspanins, such as CD9 and CD63, are also associated with protein sorting, possibly owing to their localization.

## 3 ESCRT machinery cooperates with ubiquitination and ubiquitination-like PTM in sorting

Ubiquitination is one of the most familiar PTMs, effectuating accurate alteration of translated proteins and connect a small polypeptide chain through an isopeptide called ubiquitin. This serves to regulate the function, structure, sorting, or subcellular localization of proteins, which maybe significant for the packaging of proteins to exosomes [19]. Ubiquitin connects to the proteins through the action of three enzymes, namely, E1: ubiquitin activating enzyme, which mainly activates the ATP-dependent ubiquitin; E2: ubiquitin conjugating enzyme, which combines with the ubiquitin transferred from E1; E3: ubiquitin-protein ligase, which catalyzes the binding of ubiquitin and target proteins, and E3, which can also decide the substrate specificity to a great extent because of the high heterogeneity [20].

The endosomal sorting complex required for transport (ESCRT) is the most known ubiquitin-dependent mechanism responsible for sorting ubiquitinylated proteins into ILVs. There exist various of members in the ESCRT family, including ESCRT-0, ESCRT-I, ESCRT-II, ESCRT-III, and the ESCRT-associated protein, such as Alix, which all participate in the regulation of both exosomal biogenesis and protein cargo loading. The ESCRT-dependent pathway is triggered when the ubiquitinylated membrane protein appears in early endosomes, after which the ESCRT complexes take action. ESCRT complexes work cooperatively to sort ubiquitinylated protein into exosomes: ESCRT-0 complexes consist of Hrs and STAM1/2, can recognize ubiquitinylated proteins as they possess various ubiquitin-binding domains (UBDs), and then be recruited to endosomal membranes through the combination of the Hrs FYVE domain with phosphatidyl inositol 3-phosphate (PtdIns3P). Hrs and STAM1/2 both are ubiquitinylated, thus boosting the protein cargo sorting. ESCRT-0 then recruits ESCRT-I by the binding of its PSAP-like motifs with the UEV domain of ESCRT-I, and then ESCRT-I recruits ESCRT-II subunits to collapse the endosomal membranes. ESCRT-II triggers and recruits ESCRT-III subunit Snf7 to form a ‘spiral filamentous structure’, to accelerate the endosome membrane collapse and vesicle budding. Finally, the ubiquitinylated protein is guided to the degradation, and ATPase Vps4 will also degraded so as to provide the energy that ESCRT-III required to be dissociated from the membrane [21,22], suggesting that the ending of the entrance of ubiquitinylated protein into exosomes and each subunit can be reused.

However, ubiquitination is not the only way for proteins to interact with ESCRT complexes. Some proteins were found to possess ubiquitin-like sequences, called ‘UBL-domains’, and can also work as PTM factors to regulate protein sorting, such as small ubiquitin-like modifiers (SUMO) and Ubiquitin-like 3 (UBL3). The neuronal precursor cell expressed developmentally downregulated 4 (Nedd4), among others [23]. SUMOylation indicates the attachment of a ubiquitin-like modifier to proteins so as to regulate protein-protein interactions [24]. It is consistently related to miRNA sorting to exosomes, and actually it is also found to regulate the sorting of SUMOylated proteins via the ESCRT-dependent pathway. For example, α-synuclein is the major protein of pathological aggregates of Parkinson’s Disease, which was found to co-localize with extracellular vesicles in the pullets after the ultracentrifugation. The trypsin digestion experiment demonstrated that the majority of α-synuclein recycled from pullets were enclosed by EVs instead of existing on the surface, suggesting the mechanisms of α-synuclein sorting into exosomes, and the deficiency of SUMOylation significantly shows reduction of the level of α-synuclein within EVs. Further studies showed that the SUMOylation of proteins can increase their release into exosomes via an ESCRT-dependent approach, and the recruitment of SUMO to ESCRT formation sites requires interaction with phosphoinositols and some subunits of ESCRT complexes, such as ALIX, VPS4, etc [25,26]. The vesicular transportation of α-synuclein from human cerebrospinal fluids plays an essential role in the therapy of PD, where SUMOylation functions as the sorting signal. Besides SUMOylation, there is also a protein that possesses an ubiquitin-like sequence, referred to as UBL3 protein. Researchers demonstrated that UBL3 can work as a PTM molecule to modify specific proteins through the C-terminal cysteine residue disulfide bond and facilitate proteins loading into sEVs, such as exosomes [27]. Proteomics showed that about 1241 proteins can interact with UBL3, which are called UBL3-interacting proteins and associated with at least 22 diseases. Among these proteins, Ras family members belong to proto-oncogenes, which are enriched in previously reported sEVs [28]. Hence, researchers choose Ras to perform further research. Both the wild type Ras or the mutant RasG12V can be sorted into sEVs after the UBL3 modification, and the sorting of RasG12V to sEVs even could activate Ras signaling in receipt cells by affecting ERK phosphorylation [27]. Thus, inhibiting the UBL3 modification may serve as a novel therapeutic method for sEVs-related diseases. Furthermore, Nedd4 family proteins, including Nedd4, Nedd4-2, and Itch, are thought to be members of the ubiquitin-protein ligases family, which are also essential for the trafficking of proteins [29]. Nedd4 family interacting protein 1 (Ndfip1), one of Nedd4 adaptors, binds to the protein ubiquitinylated by Nedd4, which may depend on the WW domains of Nedd4 family member and PY motifs of regulatory proteins. Researchers demonstrated that the protein secretion via exosomes and the level of protein ubiquitination in exosomes can be increased through the enhancement of the Ndfip1 level, and the knockout of Ndfip1 in cells results in the reduction of ubiquitinylated proteins in exosomes [20,30]. Furthermore, Nedd4, Nedd4-2, and Itch are not detected inside exosomes, but the co-transfection of Ndfip1 with any of these proteins could recruit these proteins into them, suggesting the function of Ndfip1 in sorting Ndfip1-binding proteins to exosomes [30]. The Nedd4 and Ndfip1 mediated ubiquitination may make sense in enhancing neuronal survival for the exosomal sorting of harmful proteins, which can promote the entrance of metal cations to the extracellular place. As another adaptor of the Nedd4 family, arrested-domain containing protein 1(Arrdc1) was always thought to associate with regulating the release of EVs (including exosomes and ectosomes) and now is considered to also regulate the sorting of protein cargos, because proteomic analysis showed that some proteins regarding apoptotic cleavage of the cell-adhesion proteins are reduced in exosomes via the knockout of arrdc1 [31].

### 3.1 Role of ESCRT subunits in sorting

The subunits of ESCRT complexes such as Alix, charged multivesicular body protein 4(CHMP4), and VPS4 also participate in the sorting of specific proteins via the ubiquitinylated-independent pathway. For instance, Alix, which works as the ESCRT accessory protein, takes action by directly binding with cytoplasmic domain to play an essential part in the protein sorting. Alix was demonstrated to interact with the quasi-enveloped HAV (eHAV) structure protein pX, which exists merely on the superficial of virus capsid of eHAV [32], so as to promote eGFP-pX sorting into exosome-like vesicles. The binding domain is not the traditional domain YPXnL, but a V domain of Alix, suggesting a novel loading mechanism. Further studies showed that full-length pX is not as effective as fragmented pX, because the C-terminal of pX (aa 31–71) represents the functional zone, while aa 1–30 may prevent protein sorting [33]. In general, the pX of eHAV may serve as a novel tool for the secretion of virions or some large exogenous protein into exosome-like vesicles with the help of Alix, which would be significant for therapy by functional delivery. Furthermore, Alix also contains a BRO1 domain to interact with the ESCRT-III subunits CHMP4, as well as the interaction with lysobisphosphatidic acid (LBPA) so as to recruit CHMP4 to the late endosome. This process probably mediates the sorting of tetraspanins into exosomes. Researchers also found that depletion of either Alix or ESCRT-III can moderate the secretion of tetraspanins CD9, CD63. and CD81 in exosomes [34].

The CHMP component can also cooperate with VPS4 to participate in the sorting of some specific membrane proteins, so as to respond to environmental and cellular signals. For example, the sorting of water channel aquaporin 2 (AQP2) to MVBs can occur to regulate water homeostasis, and in this process, lysosomal trafficking regulator-interacting protein 5 (LIP5), a small cytosolic protein acting together with the CHMP component and VPS4 seems to play a key role. Researchers found that LIP5 can interact with the CHMP component and ATPase VPS4, thus sorting the AQP2 into MVBs, which then fuses with lysosomes or the plasma membrane to be secreted in urine in the form of exosomes. LIP5 can bind to the ESCRT-III subunit CHMP1B or CHMP5 to play different roles [35]. CHMP1B binds to the MIT1 domain to recruit LIP5 to the MVB limiting membrane, and CHMP5 binds to the MIT2 domain to change the conformational change of LIP5 to stimulate Vps4 activity. The most important aspect is that LIP5 also interacts with AQP2 or other membrane protein cargos, and a series of experiments were used to prove that LIP5 binds AQP2 through the N-terminal, while AQP2 binds LIP5 through subterminal part of C-terminus. The AQP2-LIP5 complex provides a novel approach to understand membrane protein sorting [36], and it is meaningful for downregulating AQP2 during the urine volume regulation.

Furthermore,Vps4A is a subunit of Vps4 that may facilitate the sorting of β-catenin into exosomes through the interaction with β-catenin and CHMP4B. The amount of β-catenin was indeed an increase in exosomes under the condition of the overexpression of Vps4A, though the size and number of exosomes are unchanged and the colocalization of β-catenin and GFP-Vps4A also demonstrated their interaction. The silence of both CHMP4B and Vps4A decrease the level of exosomal β-catenin, thus dampening β-catenin signing so as to prevent epithelial–mesenchymal transition (EMT) in hepatocellular carcinoma (HCC) [37]. EMT is known to associated with cell metastasis, cell invasion [38], and β-catenin was demonstrated to regulate the EMT of cancer cells owing to its localization [39]. Thus, exosomal β-catenin could be used to predict the prognosis of HCC patients because of the higher level of exosomal β-catenin that the metastasis patients own, and VPS4A could regulate the localization of β-catenin, which may work as a target to treat HCC.

### 3.2 Role of lipid molecular in sorting

Although ESCRT and ubiquitination or ubiquitination-like pathways play crucial roles in protein sorting, other ways exist, as indicated by studies showing that the lack of ESCRT complexes or non-ubiquitinylated proteins only leads to a decrease of the exosomal proteins, rather than total depletion [40]. Lipid rafts are enriched in cholesterol and sphingolipids and exist on the plasma membrane. They are known as microdomains which carry functional proteins, and are found to mainly exist in the detergent-resistant membrane (DRM) of the superficial layer of exosomes [41]. They can be isolated, owing to both their insolubility in nonionic detergents and low density on sucrose density gradients [42]. The transfer of proteins to lipid rafts probably promotes the formation of early endosomes, because the mechanism called endocytosis is thought to be the invagination of the plasma membrane that is mediated by the lipid rafts. Moreover, Gassart et al. demonstrated the presence of lipid rafts in vesicles for similar cholesterols-phospholipid ratios with the exosomal plasma membrane, reaching a conclusion that lipid raft domains function as a sorting platform for ubiquitin-based protein sorting into exosomes. For example, some proteins that own an intrinstic affinity with lipid rafts, such as stomatin and flottlin-1, can be sorted into various types of exosomes. Other lipoproteins may be associated with lipid raft components, so as to be transferred into exosomes. Therefore, the tendency of protein moving to the raft domain may be related to protein sorting to exosomes [41].

Trajkovic et al. also suggested that there is an alternative pathway for sorting cargo into multivesicular endosomes, which is dependent on raft-based microdomains for segregating cargo within the endosomal membrane. These microdomains may contain high concentrations of sphingolipids, from which ceramides are formed by sphingomyelinases. After downregulating Neutral Sphingomyelinase 2 with RNAi to reduce the level of ceramide, the proteolipid protein with exosomes and within the endosomal lumen was markedly reduced. This indicates that some cargos could be transferred of exosome-associated domains into the lumen of the endosome by the sphingolipid ceramide, but not ESCRT machinery [43].

### 3.3 Role of tetraspanins in sorting

The tetraspanins also play a role in sorting. Jean-Michel et al. demonstrated that various tetraspanins, including CD37, CD53, CD63, CD81, and CD82 are enriched in exosomes released by human B-lymphocytes [44]. CD63 are commonly thought to be a marker of exosomes and are associated with protein loading and biogenesis of exosomes for its completed location patterns. Hence, CD63 is enriched on the intraluminal vesicles in late endosomes, and these vesicles are released as exosomes after endosomes fusing with the plasma membrane [45]. Based on this, the role of CD63 in exosomal protein trafficking was investigated. For example, latent membrane protein 1(LMP1) is a oncoprotein encoded by the Epstein-Barr Virus (EBV), and transporting the LMP1 into the EVs may play essential roles in virus replication, immunosuppression, or pathogenesis of EBV-associated diseases. It was reported LMP1 is copurified with CD63, and researchers have demonstrated that the increase in CD63 can cause the augmentation of LMP1 in exosomes. LMP1 reversibely increases the secretion of CD63-positive sEVs, furthermore, the knockdown of CD63 in cells could cause the reduction of LMP1 in sEVs [46], suggesting that CD63 may be associated with LMP1 sorting into sEVs, which supplies novel therapeutic targets to treat the EBV-associated cancers. In contrast, CD63 can also play a negative role. Researchers found that Herpes simplex virus 1 (HSV-1) infected cells secret more CD63-positive EVs, and in turn inhibit the virus replication, thus reducing the virus factors in the EVs [47].

Furthermore, CD63 and tetraspanin CD9 also play a key role in sorting specific proteins, such as metalloprotease CD10, which is known as the zinc-dependent membranes protease and found to be the new companion of CD9. Researchers demonstrated the interaction between CD9 and CD10 through the C-terminal tail or large extracellular loop (LEL) of CD9. Furthermore, the augment of CD9 level can respectively cause the increase of CD10 sorting into exosomes, suggesting that CD9 can recruit metalloprotease CD10 into exosomes through binding with it. Moreover, CD9 is also supposed to recruit protease molecules into exosomes to indirectly strengthen the enzymatic activity of CD10 [48]. Because CD9 and CD10 were thought to be the markers of B cell maturation [49], as well as the enhancement of the enzymatic activity of exosomal CD10 with the help of CD9, they will contribute to the modification of the extracellular matrix microenvironment and the enhancement of hematopoietic regulation of B cells.

## 4. Nuclear acids

Exosomes also contain various nuclear acids, covering mRNAs, DNAs, and non-coding RNAs, such as miRNAs [50], which can be carried to the receptor cells where these specific RNAs can take action in some scenarios [51]. For instance, EBV-miRNAs are protected from being degraded by RNase in exosomes, and are delivered to recipient cells to perform their function [52]. The non-coding RNA in exosomes can promote the metastasis of some cancers [53] or cause canceration of normal cells [54], and even act as a biomarker for the cancer [55]. The MCF7 breast cancer cell derived exosomes are found to contain the mutant DNA and mRNA. Moreover, these exosomal cargo could be incorporated into heterologous cells through MCF7 exosomes [56]. The significance of this transfer in the tumor progression must be further investigated. Furthermore, exosomal mRNAs are also thought to play a crucial role in some cases. Researchers found that exosomal mRNAs can be transferred to a target cell and then be translated into protein. Interestingly, the function of proteins translated by fragmented mRNA or full-length mRNA seems different, for example, exosomal mRNA fragmentation derived from the 3′-ends of mRNA may work as competing RNA to modulate stability, localization, and translation activity of mRNAs in recipient cells, while exosomal intact mRNA could be translated into proteins in recipient cells to change the recipient cell protein production [57].

### 4.1 miRNA loading mechanisms

MiRNAs are 17–24 nt small, endogenous, non-coding nucleotides that regulate gene expression post-transcriptionally by binding to the 3-untranslated region (UTR) or open reading frame (ORF) region of target mRNAs [58]. By comparing the level of miRNA between a subset of parental cells and their relevant derived exosomes, Gudruric-Fuchs et al. concluded that some specific miRNA, such as miR-142-3p, miR-451, and miR-150 own a higher expression level in exosomes [59]. The miRNA transferred from donor to receptor cells via exosomes indeed plays an important role in cell-to-cell communication [60]. Considering the enrichment of some miRNAs in exosomes released by multiple cell types, there must be several miRNA loading mechanisms that sort specific miRNAs into the exosomes. miRNA loading into exosomes is not sufficiently clear to date, albeit there are some hypotheses about the mechanisms of RNA sorting, such as RNA-binding proteins, the KRAS status, neutral sphingomyelinase 2, etc. to facilitate miRNAs sorting into exosomes.

### 4.2 RNA-binding proteins heterogeneous nuclear ribonucleoproteins (hnRNPs)

Heterogeneous nuclear ribonucleoproteins (hnRNPs) are a group of RNA-binding proteins that includes nearly 30 proteins which bind to nucleic acids. They play a variety of roles in regulating post-transcriptional and transcriptional gene expression, including RNA splicing, modification, localization, translation, etc. Each hnRNP possesses at least one RNA-binding domain (RBD). HnRNPA2B1 is one of the most common hnRNPs, which may regulate the sorting of specific miRNAs. Some miRNAs that can be packaged into exosomes are called EXO miRNA (miR-198, miR-601, etc.) compared with some miRNAs (miR-17, miR-29a, etc.) that just remain in cells (CL miRNA). The motifs in EXOmiRNA and CLmiRNA are quite different, suggesting the importance of the sequence in miRNA sorting mechanism. The silence of hnRNPA2B1 or mutant of specific EXO-motifs can cause a decrease in EXOmiR-198 rather than other CLmiRNAs, indicating the function of hnRNPA2B1 in miRNA sorting. hnRNPA2B1 may mediate the loading of EXOmiRNA, such as miR198 and miR601, through binding to some special EXO-motifs, such as GGAG [61]. Another study on the crystal structures of hnRNP A2B1 identified that the RRM1 and RRM2 domains of hnRNPA2B1 specifically recognize AGG and UAG motifs, respectively [62]. SUMOylation is parallel to ubiquitination, which is also a post-translational modification involved in numerous biological processes, such as carcinogenesis, DNA damage repair, etc [63]. Researchers demonstrated that hnRNPA2B1 must be SUMOylated before binding to EXO motifs. HnRNPA2B1 possesses an affinity to the raft-like region [61], and it is well known that exosomes originate from the inward budding of the endosomal membrane. Thus, ILVs, which then enter the MVBs, such that the protein can first bind with EXOmotifs of some miRNAs and then to the ceramide-rich membrane region in the cytoplasmic leaflet of MVB membrane to package miRNAs to the exosome [61]. The HnRNPA2B1 mediated miRNA sorting into exosomes may be a novel method for the cell-to-cell communication, or just remove some unwanted miRNA to the extracellular space. Importantly, it may offer novel insight for the artificial loading of some miRNAs with specific EXOmitifs for gene therapy. Furthermore, miR-503 can also be loaded into exosomes derived from endothelial cells. Although miR-503 can interact with hnRNPA2B1, it does not possess the EXO motifs as described previously. This suggests that the binding of hnRNPA2B1 with miR-503 follows different rules, and the study shows that the knockdown of hnRNPA2B1 interestingly increases the sorting of miR-503 into exosomes and weakens the exosomal packaging of miR-503, reaching a conclusion that hnRNPA2B1 can also negatively regulate the sorting of specific miRNAs [64], which offers a novel mechanism of the inhibition of exosomal miRNA transport to extracellular space to promote cellular retention. Besides hnRNPA2B1, two other hnRNP proteins, called hnRNPA1 and hnRNPC, may exist that participate in the process of the sorting of exosomal miRNA by binding to specific motifs. However, researchers failed to find out what the exact motifs are [40].

### 4.3 Synaptotagmin-binding cytoplasmic RNA-interacting protein (SYNCRIP)

Synaptotagmin-binding cytoplasmic RNA-interacting protein (SYNCRIP) is an RNA-binding protein that exists in extracellular vesicles derived from multiple cell types. It is also a component of the Hepatocyte Exosomal Machinery [6]. Laura Santangelo et al. [65] used western blot analysis to observe that SYNCRIP can specifically bind to miR-3470a and miR-142-2-3p, which are both proved to be the exosomal-enriched miRNA (hEXO-miRNAs). Researchers also found that hEXO-miRNAs all share a common sequence GGCU, which is demonstrated to be the SYNCRIP binding motif and may regulate the localization of miRNA because the insertion of the hEXO-motif to natural cell-retained miRNAs can cause a transportation into exosomes. Furthermore, the deficiency of SYNCRIP or the knockdown of the GGCU motif can both decrease the expression level of hEXO-miRNAs. These findings provide novel insight on the sorting of miRNAs on the role of SYNCRIP, which may be helpful for understanding of the cell-to-cell communication reliance on hepatocytes vesicle-associated miRNA.

### 4.4 Y-box binding protein 1(YBX-1)

The Y-box binding protein 1(YBX-1) can be detected in isolated exosomes. It functions as the RNA-/DNA-binding multifunctional protein that participates in numerous processes, such as RNA stabilization, transcription regulation, DNA repair, etc. [66]. Researchers demonstrated that YBX-1 may act as an exosomal miRNA sorting regulator to sort miR-233 into exosome through binding with it even in a cell-free reaction [67]. The exosomal miR-233 can be transferred to macrophages to promote their differentiation [68]. Moreover, besides miR-233, miR-133 is also found to be packaged into hypoxia/reoxygenation induced endothelial progenitor cell (EPC)-derived exosomes by YBX-1, and these exosomes with enriched miR-133 could regulates fibroblast angiogenesis and the mesenchymal–endothelial transition (MEndoT) [69]. The recognized RNA binding motif is CAUC, as previously demonstrated [70].

### 4.5 Major vault protein (MVP)

The major vault protein (MVP) is also a specific RNA-binding protein that mainly binds with miR-193a. MiR-193a is known as a regulator of colon cancer progression, and researchers showed that MVP indeed can interact with miR-193a via immunoprecipitation. Moreover, the knockout of MVP shows an increase of the cellular miR-193a expression, and inversely a decrease in the exosomal miR-193a [71]. These studies confirm the ability of MVP to mediate miR-193a sorting into exosomes to accelerate colon cancer progression.

### 4.6 Fragile X mental retardation 1 (FMR1)

Fragile X mental retardation 1 (FMR1) is also kind of a RBP, which is known to be associated with the trafficking of RNA with the help of kinesin and exerts transcriptional repression. FMR1 is also found enriched in exosomes released during inflammatory processes [72,73]. The release of exosomes maybe a way for responding to some inflammatory diseases, and these exosomes own abundant variable cargos of proteins and RNA [74,75]. Recently, researchers revealed the role of RBP FMR1 in exosomal miRNA loading during inflammation, and found that the cleaved Rab-interacting lysosomal protein (RILP), which is a Rab7 trafficking adaptor protein, can alter vesicle trafficking [76], and increase the release rate of exosomes, as well as interact with FMR1 to alter the level of specific miRNA that contain AAUGC motifs like miR-155. In this process, one of the ESCRT III protein- Hrs, also participates through binding with FMR1 [77]. This study provides a better understanding of exosomal trafficking during the inflammation, and offers novel insight for the diagnosis and treatment of inflammation-related diseases.

### 4.7 Argonaute 2 (Ago2)

Argonaute 2 (Ago2) is a component of RNA-induced silencing complex (RISC) that regulates the abundance of miRNA after transcription. The decrease of endogenous Ago2 will reduce the expression and activity of mature miRNA [78]. Further, Ago2 was also found to exist inside exosomes, and work as an RNA binding protein to bind with some exosomal miRNAs to form an Ago-miRNA complex, thus regulating miRNA secretion in exosomes. Studies demonstrated that the knockdown of Ago2 can decrease the level of some specific exosomal miRNAs, such as miR-100, let-7a, so the level of Ago2 is essential in miRNA sorting [79]. However, an oncogene called KRAS can influence the role of Ago2. Studies showed that mutant KRAS have more activation of KRAS-MEK-ERK signaling, which may prevent the Ago2-miRNA complexes sorting into exosomes, and possibly by enhancing S387 phosphorylation [79,80]. These studies identify a novel exosomal miRNA sorting regulator and implicate essential regulatory signaling mechanisms.

### 4.8 Sorting mechanism of RBPs

Having described the mechanisms of RBPs binding with specific miRNA to gain a basic understanding, a novel question arises on how the RBPs are sorted into exosomes emerged. After a series of studies, researchers use proteomics and RNA analysis of EVs demonstrating that MAP1LC3B(LC3)-conjugation machinery is needed for the packaging and secretion of RBPs or small non-coding RNA. LC3 is one of the autophagy proteins that can interact with RBPs and then be sorted into LC3 enriched exosomes for secretion outside cells. This can also be described as a secretory autophagy pathway [81,82].

## 5. Neutral sphingomyelinase 2-dependent mechanism

Neutral Sphingomyelinase 2 (nSMase2) can stimulate budding of the endosomal membrane to modulate the secretion of exosomes and regulate the release of exosomal miRNAs. These miRNA can be transferred to the receptor cells to promote cell-to-cell communication [83]. For example, exosomal miR-210 derived from metastatic cancer cells is nSMase2-regulated, which can be sorted into endothelial cells, resulting in its metastasis efficiency by suppressing some target genes [84], which may offer a novel therapeutic method for the cancer by inhibiting angiogenesis though exosomal miRNA. Similarly, miR-10b from breast cancer cells MDA-MB-231 can also be transferred to HMLE cells through enveloped by exosomes, and the exosomal miR-10b still retain its function in the receptor cells, which can suppress the expression of target gene HOXD10 to promote their invasion into them [85]. Targeting the exosomal miRNA probably offers novel means for the treatment of breast cancer. Furthermore, when those cells were treated with GW4869, which is the inhibitor of nSMase2, upon analysis of the level of responding miRNA to make a comparison, results showed that the level of the miRNA both shows an increase in intracellular and a decrease in exosomes, suggesting these miRNAs are all secreted in a nSMase2-dependent way [84–86].

### 5.1 KRAS-dependent way

KRAS is an oncogene brought by the rat sarcoma virus, which can be categorized into two types: wild-type (normal) or mutant (abnormal), mainly occuring in colon cancer with the probability of about 34–45%. Currently, research showed that KRAS status may influence the profile of miRNA both in exosomes and their parental cells, for instance, miR-100 owns a higher expression level in mutant exosomes, while miR-10b expresses more in wild-type exosomes, and miR-320 families are enriched in both mutant and wild-type KRAS exosomes, suggesting miR-320 families may sorted into exosomes, regardless of the KRAS type [86]. The different miRNAs associated with different KARS status imply the possibility of exosomes to be the biomarker of CRC, and suppressing retention or transportation of some miRNAs may treat the cancer caused by aberrant miRNA expression. Interestingly, through analyzing two strands of miR-423, it is suggested that miR-423-5p are overexpressed in wild-type KRAS instead of mutant KRAS exosome and corresponding cells. Hence, the KRAS type may also influence the guide or passenger chain to select specific miRNAs into exosomes.

### 5.2 mRNA loading mechanisms

Besides miRNA, exosomal cargos also include mRNAs, which may provide a novel insight in cancer diagnostics and therapies and promote cell-to-cell communication [87]. Similarly, there still exist specific mechanisms about the sorting of mRNA into exosomes because of the selective enrichment of mRNA in exosomes. Researchers found that the 3ʹ-untranslated region (UTR) fragments of mRNA are enriched in MVs (including exosomes) which may explain the loading mechanism. Zipcode, a sequence in the 3ʹUTR of mRNA transcripts regulate the binding of a ribonuclear protein complex with mRNA, thus influencing the blockade of translation and the location of mRNA. mRNA will then move to a cellular location to restart release and translation [88]. The zipcode-like 25 nucleotide sequence is kind of specific Zipcode, which owns the CTGCC core domain and a miRNA-binding site. When the zipcode-like sequence is incorporated into 3ʹUTR fragments of mRNA, the amount of this mRNA in MVs including exosomes and microparticles increased, and the appearance of both elements in the enriched mRNA in MVs are more than twice compared with those non-enriched mRNAs. The silence of the ‘CTGCC’ sequence can cause a decrease of mRNA within the MVs, suggesting the essential role of the sequence in the sorting of mRNA. Furthermore, the miRNA binding site of 25-nt zipcode binds directly with miR-1289. Mizrak et al. showed that the increase of miR-1289 also enhances the level of mRNA in MVs, indicating the level of miRNA may also counts. In summary, the cooperation of CTGCC core domain and miRNA-binding site promotes the loading of mRNA into MVs [89]. The mRNA inside the MVs can be transferred to the receipt cells to translate into proteins to take action. Thus, incorporating the zipcode-like sequence into some therapeutic RNAs to sort them into MVs may serve as a therapeutic tool by transferring them in to the receptor cells.

YB-1 is always linked with the sorting of miR-133 and miR-223 as previously discussed [67,69]. However, it also makes sense in mRNA loading into exosomes. Researchers used a model septin 14 mRNA (SEPT14 RNA), which mimics the 3ʹUTR fragments of exosomal mRNA that contains three eRNA-specific motifs: (1) ACCAGCCU, (2) UAAUCCCA, and (3) CAGUGAGC. These motifs are thought to work as a regulator to target the RNAs into exosomes. YB-1 was found to recognize and bind to these motifs with different affinity, and this connection is assisted by the C-terminal of YB-1, suggesting the essential role of YB-1 to load mRNA into exosomes [90]. Understanding this mechanism maybe helpful for loading other functional mRNAs into exosomes for treatment or diagnosis.

### 5.3 gDNA loading mechanisms

The other type of cargo in EVs is DNA, the significance of the existence of extracellular DNA may be protect the degradation of DNA from nucleases, which are present in circulation [91]. By using iodixanol density gradient separation, the subtype of sEVs was separated, consisting of the high density (HD) fraction, which contain the most sEVs associated DNA, and low density (LD) fraction, which include little DNA but the more classic sEVs. However, majority of the DNA exists on the surface of sEVs, while it was found that the genomic DNA (gDNA) and mtDNA may exist inside the sEVs, which can protect itfrom digestion by DNase [92]. Now, we emphatically address the gDNA sorting mechanisms, as exosomal gDNA was derived from cancer cell instead healthy cells, which mediates cellular senescence and stimulation [93,94]. gDNA principally exists in the cell nucleus, which does not exhibit any interaction with cytoplasmic MVBs [95]. Hence, the presence of gDNA in the exosomes seemed unexplainable before a novel mechanism was discovered. The micronuclei (MN) is a cytoplasmic structure encased in nuclear membrane, and the formation of MN can be a marker of genomic instability, because it is released when the cell nucleus is unstable [96]. Similarly, the envelope of MN is also unstable and can be break down under the situation of cell division and release the inside contents, such as gDNA. Upon these studies, researchers demonstrated the relationship between MN and nEXO (exosomes that contain gDNA and other nuclear contents), MN and gDNA both present in cancer-derived exosomes, on account of the instability of the MN envelope, nuclear membrane can effortlessly collapsed to release its nuclear contents, once it does, the MVB which situated near early endosome and MN will interact with the collapsing MN, and tetraspanin CD63 soon surround the disrupted MN to form a gDNA-nuclear proteins complex. Researchers also found that the knockdown of CD63 can decrease the amount of nEXOs via flow cytometry, indicating the function of CD63 in nEXOs sorting [94]. In short, tetraspanin CD63 surround nuclear contents released by disrupted MN and load them into the exosomes, and nEXO may provide a novel insight for the cancer biomarker development.

## 6. Lipid cargo

Lipids are fundamental components of exosomal membranes. Compared with parent cells, the contents of some lipids are quite different. For example, sphingomyelin, caramide, phosphatidylserine, and glycosphingolipids are 2 ~ 3 times richer in exosomes than parent cells, while phosphatidylcholine (PC) is less in exosomes than cells, and the difference of phosphatidyl ethanolamine (PE) between exosomes and cells is insignificant [97]. There is not sufficient research about the sorting mechanism of lipids; however, some researchers believe that the sorting mechanism of lipid has no relationship with that of protein. U87, Huh7, and MSC are used to test the hypotheses. Proteomics and lipidomic showed that protein not lipid sorting mechanisms into EVs depended on the cell type, while lipid sorting into EVs was related to the size and quantity of exosomes [98]. Furthermore, the role of the myelin proteolipid protein (PLP) in lipid sorting was initially studied, the incorporation of PLP in to EVs is in dependent of ESCRT mechanisms [43]. Researchers found that PLP-existing EVs showed an enrichment of majority of lipids including ceramide, which is known to formed by the activation of sphingomyelin hydrolysis by nSMase2 [99]. Moreover, the inhibition of nSMase2 could dampen the secretion and biogenesis of PLP-containing EVs, which may reversely suppress the expression of lipid in EVs. The research about the lipid sorting mechanisms is limited, and waiting to be discovered.

## 7. Discussion

Exosomes can work as carriers to carry cargos, such as various proteins and RNAs, to the receptor cells. The exosomal cargos can be extensively influenced by the cellular state and provide a reflection on the changes of cellular microenvironment [19]. Thus, analyzing these cargos may be helpful for the clinical diagnosis and therapy. However, cargo sorting mechanisms of exosomes are very complex processes that are involved in numerous participants and regulators. Ubiquitination and the ESCRT-dependent approach are thought to be the most essential way of protein sorting, even in the biogenesis of exosomes, while the ESCRT-independent approach, like lipids and tetraspanins also are reasonable. RNA sorting mostly depends on the RNA-binding proteins, such as SUMOylated hnRNPA2B1 that can bind with specific miRNAs through some short sequence motifs exist in the miRNAs. However, not all miRNAs possess motifs, indicating another sorting mechanisms such as nSMase2, KRAS dependent ways, and specific structure or miRNA themselves, like miR-1289 can promote the sorting of mRNA into exosomes, etc. With regard for the extraction methods of sEVs, the most classic and the common method is differential ultracentrifugation, and the majority of articles have used this method to extract sEVs. Although there may exist minor different in the centrifugal force, their principles are quite similar, and the small centrifugal force (e.g.,300 g) removes dead cells, while the medium centrifugal force (e.g. 2000 g) removes cell debris, followed by large centrifugal force (e.g. 10,000 g) to remove large MVs. Finally, the overspeed centrifugal force (e.g.,100,000 g) is able to pellet the sEVs, and this method we called ‘1ʹ in Table 1. However, sEVs extracted by method 1 may be not pure, and under this circumstance, other methods may be used after the method 1 to purify sEVs, such as sucrose/iodixanol gradient centrifugation used in some articles [41,46,61,67,71,100]. Sucrose or iodixanol is used to form a continuous or discontinuous density gradient in the centrifugal tube, and samples of different densities (sEVs and non-vesicle particles) are stratified and separated by the centrifugal force [101]. This method, which we call ‘method 2ʹ in Table 1, could obtain comparatively pure sEVs, and it is widely used in experiments that must separate the subtypes [100]. Furthermore, purification of sEVs could also use corresponding immunomagnetic beads, which we call ‘method 3ʹ, such as exosomal marker CD63 [67]. This method is more specific, and the operation is simple; but the regent is expensive, and it is not suitable to obtain exosomes from large numbers of samples. Size-exclusion chromatography (SEC) achieves separation based on size rather than molecular weight, the technique uses columns filled with porous polymer microspheres, where molecules pass through the microspheres according to their diameters [102]. Smaller molecules take longer to migrate through the column’s pores, while larger molecules are eluded earlier from the column. We define size exclusion chromatography as ‘method 4ʹ, which can accurately separate large and small molecules, but this method takes a long time and is not suitable for processing a large number of samples. Polyethylene glycol precipitation such as exoquick we called ‘method 5ʹ also used in some articles [37] can quickly isolate sEVs without the overspeed centrifugal force. However, these methods may isolate non-vesicular contaminants (including lipoproteins) at the same time, and the confounding of polymer-based materials may affect downstream analysis. Different extraction methods must be selected according to the different experimental purposes, and possibly the mixture of some methods can achieve better consequences.

**Table 1.** List of cargos of sEVs sorting mechanisms

Type ofcargo	Sorted cargo	Related molecules	Sorting mechanism		Role of exosomes/Related disease/miRNA Binding Motifs	Extraction Methods for sEVs	Ref.
**protein**	Nedd4 familyproteins	Ndfip1	ubiquitination-like	Ndfip1 interacts with Nedd4 family proteins	Remove the harmful substances to improve neuronal survival in case of brain injury.	1	[20, 30]
	α-synuclein	SUMO		SUMOylation of proteins via interacting with phosphoinositols and some subunits of ESCRT complex such as ALIX, VPS4.	Helpful for analyzing the Parkinson’s Disease pathogenesis at molecular level.	1 and 2	[25,26]
	RasG12V	UBL3		UBL modify proteins through the C-terminal cysteine residue disulfide bond.	Sorting of RasG12V could improve the activation of Ras signaling.	1	[27]
	eGFP	pX, Alix	ESCRT subunits	C-terminal of Px interacts with V domain of Alix to sort the eGFPpX into exosomes.	Promotes secretion of virions and foreign proteins through exosomes.	1	[33]
	AQP2	LIP5, Vps4		LIP5 binds with AQP2, and CHMP1B recruit LIP5 to MVBs, which then fuse with plasma membrane to release as exosomes.	Transfer specific membrane proteins so as to respond to cellular and environmental signals.	/	[36]
	β-catenin	Vps4A, CHMP4B		Vps4A may facilitate the sorting of β-catenin into exosomes through the interaction with β-catenin and CHMP4B.	Prevent epithelial-mesenchymal transition (EMT) in hepatocellular carcinoma by dampening β-catenin signing	1 and 5	[37]
	Stomatin flottlin-1	lipid rafts	lipid and tetraspanins	Lipoproteins associated with lipid raft components	Emphasizing the existence of lipidmicrodomains in exosomal membranes and the direct impact of exosomes in regulatory mechanisms	1 and 2	[40,41]
	LMP1	CD63		Copurification of CD63 for its completed location patterns	LMP1-modified exosomes show the ability to enhance progression of EBV-associated cancers.	1 and 2	[46]
	CD10	CD9		CD9 interacts with CD10 via establishing the chimeras	Release of CD10 peptidase activity with exosomes may effectively regulate extracellular matrix microenvironments	1	[48]
**RNA**	miR-198, miR-601	hnRNPA2B1	RNA Binding Proteins	SUMOylation of hnRNPA2B1 bind with GGAG of some miRNAs.	GGAG motif	1 and 2	[61]
	miR-3470a, miR-194	SYNCRIP		RBPs bind with motifs of specific miRNAs	GGCU motif	1	[65]
	miR-233, miR-133	YBX-1			CAUC motif	1 and 2 and 3	[66,67,69]
	miR-193	MVP			NA	1 and 2	[71]
	miR-155	FMR1			AAUGC motif	1	[77]
	miR-10b, let-7a	Ago2		Ago2 bind with miRNA to form an Ago2-miRNA complex	NA	1 and 2	[79–86]
	miR-10b, miR-100	KRAS,	KRAS status and nSMase2 influence the profile and the level of miRNA.		Change of miRNA expression may play therapeutic roles in reversing the tumorigenic effects on account of the aberrant miRNA expression.	1	[86]
	miR-210 miR-10b	nSMase2	Release of exosomes and exosomal miRNA are regulated by nSMase2.				[84, 85]
	mRNA	CTGCC core domain, miRNA-binding site	Zipcode-like 25 nucleotide sequence which contain CTGCC core domain and miRNA-binding site work as a Zipcode exist in the 3’UTR of mRNA to promote the sorting.		Can be a potential approach for cancer gene therapy by incorporating sequences into 3’UTR of therapeutic RNAs.	1	[89]
		YB-1	RNA Binding Proteins	YB-1 recognize and bind to eRNA-specific motifs with different affinity, and this connection is assisted by the C-terminal of YB-1.	(1) ACCAGCCU (2) UAAUCCCA (3) CAGUGAGC	1	[90]
**DNA**	gDNA	Micronuclei (MN), Tetraspanins	CD63 surrounded gDNA released by disrupted MN, and participate in the exosome biogenesis and finally be sorted into exosomes.		Highlights the function of nEXO in cancer biomarker development.	1 and 4	[94]

## 8. Conclusion

It is now clear that sEVs contains various of cargos, which can play essential roles, and diverse cargos may own different sorting mechanisms. In this article, we reviewed major mechanisms, such as ESCRT-dependent and the ESCRT-independent approach for protein sorting, the RBP-related way of majority of miRNAs sorting, and we summarized the exosomal sorting mechanisms, the biological roles of different kinds of cargos in Table 1. However, the involved mechanisms may not complete and require researchers to explore further, maybe further study of these processes could make sense in the future diagnosis and therapy of some diseases.
